# *Otx2* Requires *Lmx1b* to Control the Development of Mesodiencephalic Dopaminergic Neurons

**DOI:** 10.1371/journal.pone.0139697

**Published:** 2015-10-07

**Authors:** Orna Sherf, Limor Nashelsky Zolotov, Keren Liser, Hadas Tilleman, Vukasin M. Jovanovic, Ksenija Zega, Marin M. Jukic, Claude Brodski

**Affiliations:** Department of Physiology and Cell Biology, Zlotowski Center for Neuroscience, Faculty of Health Sciences, Ben-Gurion University of the Negev, Be’erSheva 84105, Israel; Rutgers University, UNITED STATES

## Abstract

Studying the development of mesodiencephalic dopaminergic (mdDA) neurons provides an important basis for better understanding dopamine-associated brain functions and disorders and is critical for establishing cell replacement therapy for Parkinson’s disease. The transcription factors *Otx2* and *Lmx1b* play a key role in the development of mdDA neurons. However, little is known about the genes downstream of *Otx2* and *Lmx1b* in the pathways controlling the formation of mdDA neurons *in vivo*. Here we report on our investigation of *Lmx1b* as downstream target of *Otx2* in the formation of mdDA neurons. Mouse mutants expressing *Otx2* under the control of the *En1* promoter (*En1* ^+/Otx2^) showed increased *Otx2* expression in the mid-hindbrain region, resulting in upregulation of *Lmx1b* and expansion of mdDA neurons there. In contrast, *Lmx1b* ^*-/-*^ mice showed decreased expression of *Otx2* and impairments in several aspects of mdDA neuronal formation. To study the functional interaction between *Otx2* and *Lmx1b*, we generated compound mutants in which *Otx2* expression was restored in mice lacking *Lmx1b* (*En1* ^+/Otx2^;*Lmx1b* ^*-/-*^). In these animals *Otx2* was not sufficient to rescue any of the aberrations in the formation of mdDA neurons caused by the loss of *Lmx1b*, but rescued the loss of ocular motor neurons. Gene expression studies in *Lmx1b* ^*-/-*^ embryos indicated that in these mutants *Wnt1*, *En1* and *Fgf8* expression are induced but subsequently lost in the mdDA precursor domain and the mid-hindbrain organizer in a specific, spatio-temporal manner. In summary, we demonstrate that *Otx2* critically depends on *Lmx1b* for the formation of mdDA neurons, but not for the generation of ocular motor neurons. Moreover, our data suggest that *Lmx1b* precisely maintains the expression pattern of *Wnt1*, *Fgf8* and *En1*, which are essential for mid-hindbrain organizer function and the formation of mdDA neurons.

## Introduction

Meso-diencephalic dopaminergic (mdDA) neurons modulate essential brain functions including motor control, cognition and reward. Their degeneration and dysfunction has been associated with prevalent and devastating brain disorders such as Parkinson’s disease, schizophrenia and drug abuse[1]. mdDA neurons are organized in three major nuclei: substantia nigra, ventral tegmental area and retrorubral field[1]. Characterizing the molecular mechanisms controlling the development of mdDA neurons has attracted special interest since they provide critical information for the differentiation of stem cells into DA neurons used in cell replacement therapy for Parkinson’s disease[2][3].

mdDA neurons originate from the midbrain floor plate and probably partially also from the diencephalon[4]. The morphogens, *Shh*, *Wnt1* and *Fgf8* that provide progenitors with positional information, instructing them with regard to their dopaminergic identity, are secreted from the ventral midbrain and the mid-hindbrain organizer (MHO)[4][5][6][7][8][9]. These morphogenes work in concert with a series of transcription factors that include *Otx2*, *Lmx1b*, *Lmx1a*, *En1*/2, *Foxa1*/2, *Ngn2*, *Pitx3* and *Nurr1 (Nr4a2*– Mouse Genome Informatics). Each is important for progenitor cell responsiveness to morphogens, differentiation and survival, as well as for MHO positioning and activity[4][10][11][12][13][14][15][16][17][18][19]. Thus, while many proteins involved in the development of mDA neurons have been identified, understanding how they interact to ensure proper temporal and spatial establishment of mdDA cells remains a major challenge.

Different lines of evidence indicate that *Otx2* is a key regulator of mDA development. By employing mouse mutants in which the caudal *Otx2* domain, and concomitantly the MHO, are shifted caudally or rostrally, we initially demonstrated that *Otx2* controls the number and location of mdDA neurons by positioning the MHO[10]. Subsequent work demonstrated that *Otx2* can regulate the formation of mdDA neurons during later stages of development, independent of the position of the MHO[11]. This central role for *Otx2* in the ontogeny of mdDA neurons has gained support by reports demonstrating that *Otx2* regulates the neurogenic activity in the midbrain floorplate, as well as proliferation and differentiation of mdDA progenitors[20][21][22]. Finally, overexpressing *Otx2* in the mid-hindbrain region leads to a lateral expansion of the mdDA neuronal population, indicating that *Otx2* controls the medio-lateral extension of the mdDA precursor domain[21]. Different genes are regulated in *Otx2* gain- and loss-of-function experiments, suggesting that each might mediate the effects of *Otx2* on mdDA neuron ontogenesis. Thus far, however, functional *in vivo* studies testing whether these genes indeed mediate *Otx2* effects on the development of DA neurons are lacking.

Expression of *Lmx1b*, found in both the mdDA progenitor domain and in the MHO, is regulated in chickens by *Otx2*[23]. This supports the contention that *Lmx1b* is downstream of *Otx2* in the genetic network controlling the generation of mdDA neurons. In *Lmx1b* ^*-/-*^ mutants, different aspects of mdDA development are altered. For example, caudomedial mdDA neurons, that will predominantly form the ventral tegmental area, exhibit impairments in their terminal differentiation as indicated by a reduced number of NURR1^+^ cells expressing TH, and likewise fail to express PITX3 [12][24]. Moreover, the lateral mdDA neurons, forming the bulk of the substantia nigra, are missing, as indicated by a loss of *Wnt1* and D2R expression[24]. Finally, the red nucleus (RN), located lateral to the mdDA precursor domain, inappropriately extends medially into the mdDA domain, suggesting that *Lmx1b* is critical to define the medio-lateral borders of the mdDA nuclei[24]. Another non-DA population, the ocular motor neurons (OMNs) are almost completely missing in *Lmx1b* ^*-/-*^ mutants[24].

Experiments featuring conditional activation and inactivation of *Lmx1b*, using an *En1*-Cre driver, demonstrated increased and decreased numbers of TH^+^ neurons respectively, further indicating the importance of *Lmx1b* for the formation of mdDA neurons[9]. The conditional inactivation of *Lmx1b* using a *Shh*-Cre driver suggest that *Lmx1b* is non-cell autonomously required for mdDA neuron development, indicating that the effects of *Lmx1b* on mdDA ontogeny are mediated by the MHO expression domain[25]. Since both *Wnt1* and *Fgf8* are the key mediators of MHO activity and required for mdDA development, they are the most likely candidates to mediate the MHO effects of *Lmx1b* on mdDA development. *Lmx1b* cooperates with *Wnt1* and with *miR135a2* to control the generation of mdDA neurons[26][27]. Little is known, however, about the aspects of mdDA neuronal development regulated by the *Lmx1b/Wnt1* interaction. A role for *Fgf8* in mediating *Lmx1b* activity has not been established and the data on the regulation of *Fgf8* by *Lmx1b* is inconsistent. *Fgf8* has not been detected in *Lmx1b* ^*-/-*^, suggesting that *Lmx1b* is absolutely required for *Fgf8* induction[28]. However, these findings are in conflict with the induction of medial mdDA neurons in *Lmx1b* ^*-/-*^, since previous work indicates that the *Fgf8* expression domain at the MHO is required for mdDA induction[5].

In our study, we found that expression of *Otx2* and *Lmx1b* are reciprocally regulated. We furthermore provide evidence that *Otx2* cannot compensate for the loss of *Lmx1b* in the specification of mdDA neurons, but it can replace *Lmx1b* in the induction of OMNs. This indicates that *Lmx1b* is downstream of *Otx2* in the genetic pathway controlling mdDA formation but not OMNs generation. Moreover, we performed a detailed expression analysis of *Lmx1b* ^*-/-*^ embryos and found that *Lmx1b* is not required for the induction of *Wnt1*, *En1* and *Fgf8*, but is necessary for maintaining their transcripts in a specific, spatio-temporal manner in the mdDA precursor field and MHO.

## Materials and Methods

### Animals

The generation of *En1* ^*+/Otx2*^ and *Lmx1b* ^*-/-*^ mice has been reported earlier[29][10][30]. *En1* ^*+/Otx2*^ embryos were distinguished from their wild type littermates, by genotyping PCR using primers: forward 5'-GGGCTGAGTCTGACCACTTC–3' and reverse 5' -CAGGAAGCTGGTGATGCATA–3', resulting in a 625 bp product. *Lmx1b* null allele genotyping was done by PCR using primers: forward 5′-GATAGGGCATTCAACCAGGACGAGCAAAGA and reverse 5′-AAACAGAAGCCACAGAGAGCCAAGGAGAAG, resulting in a 397bp fragment. In order to obtain embryos at a specific stage, mice were bred overnight, and females were checked the following morning for the presence of copulatory plugs. Embryos were collected at embryonic day (E) 9.5 to E12.5.

Mice were kept under standardized conditions (22–24°C temperature; 55%±15% humidity) on a 12 h light / 12 h dark cycle in groups of 3–5 in standard laboratory cages. Food and tap water were provided *ad libitum*. Mice were sacrificed by isoflurane overdose. All animal studies were performed in accordance with local animal welfare regulations and international guidelines. 42 adult animals and 87 embryos were used for the study. The protocol was approved by the Committee on the Ethics of Animal Experiments of the Ben-Gurion University of the Negev (Permit Number: IL-13-03-2010). All efforts were made to minimize suffering.

### RNA *in situ* hybridization

For section *in situ* hybridization, embryonic mice were immersion fixed with 4% paraformaldehyde (PFA), paraffin embedded and cut on a microtome in 8μm thick sections. All sections were processed for *in situ* hybridization according to Brodski et al., 2003 using 35S-labeled riboprobes. Antisense RNA probes were transcribed from plasmids containing fragments of the murine: *Otx2*, *Wnt1*, *Th*, *Fgf8*, *Lmx1a*, *En1 and Nurr1* as described in Brodski et al. 2003.

### Immunohistochemistry and Cell Counting

For fluorescence immunohistochemistry, paraffin sections (5 μm) were deparaffinized through xylene, rehydrated through an ethanol series, washed in PBS. After antigen retrieval in boiling citric acid (pH–6) for 10 minutes, sections were blocked in 4% normal goat serum (NGS) for half an hour. Slides were incubated with primary antibody diluted in PBS overnight, washed 3xPBS for 3 minutes and incubated for 1 hour with secondary antibody. Slides were washed 3xPBS for 5 minutes and mounted with Immu-Mount (Thermo Scientific). Antibodies used: rabbit anti TH (Millipore, 1:200), mouse anti TH (Millipore 1:200), rabbit anti OTX2 (Abcam, 1:300), mouse anti FOX2A (DSHB, 1:10), rabbit anti NURR1 (Santa Cruz, 1:400), rabbit anti LMX1A (Millipore 1:100), rabbit anti Cleaved CASPASE 3 (Cell Signaling, 1:300), mouse anti POU4F1 (Santa Cruz, 1:400), mouse anti NKX2.2 (DSHB, 1:50), mouse anti EN1 (DSHB, 1:50), mouse anti NKX6.1 (DSHB, 1:10), rabbit anti PHH3 (Millipore,1:1000), mouse anti LIM (DSHB, 1:50), mouse anti ISLET1 (DSHB, 1:50). Secondary antibodies: goat anti rabbit Cy3, goat anti mouse Cy3, goat anti rabbit Alexa-Fluor–488, goat anti mouse Alexa-Fluor–488, all 1:100 (Jackson Laborotories). In pictures depicting sagittal sections, the rostral part was oriented to the left. For cell counting, coronal sections (5 μm thick) of each genotype were immune stained with the relevant antibody and positive cell bodies were counted on every second section of all slides.

All results are expressed as mean ± SE. IBM^®^ SPSS^®^ Statistics 21 software was used for the statistical analysis. Genotypes were analyzed as factors for two-way multifactorial analysis of variance (ANOVA). Where significant effects were detected, Fisher’s LSD post hoc analysis was used to detect differences between individual groups (containing at least 3 animals).

## Results

### Overexpressing *Otx2* in the hindbrain leads ventrally to a specific expansion of the mdDA neuronal population

Previous studies on *En1* ^+/Otx2^ mice indicate that a caudal shift of the *Otx2* expression border (and concomitantly the MHO), leads to caudal enlargement of the entire dorsal midbrain and a complementary reduction of dorsal hindbrain[29]. In the ventral midbrain of *En1* ^+/Otx2^ mutants, the caudal shift of *Otx2* and the MHO leads to an increase in the number of mdDA neurons, but does not affect the formation of OMNs[10]. In order to study the specificity of the enlargement of the mdDA neuronal population in *En1* ^+/Otx2^ mutants, we investigated the formation of the RN, since it is the nucleus most closely neighboring mdDA nuclei.

**Fig 1:**
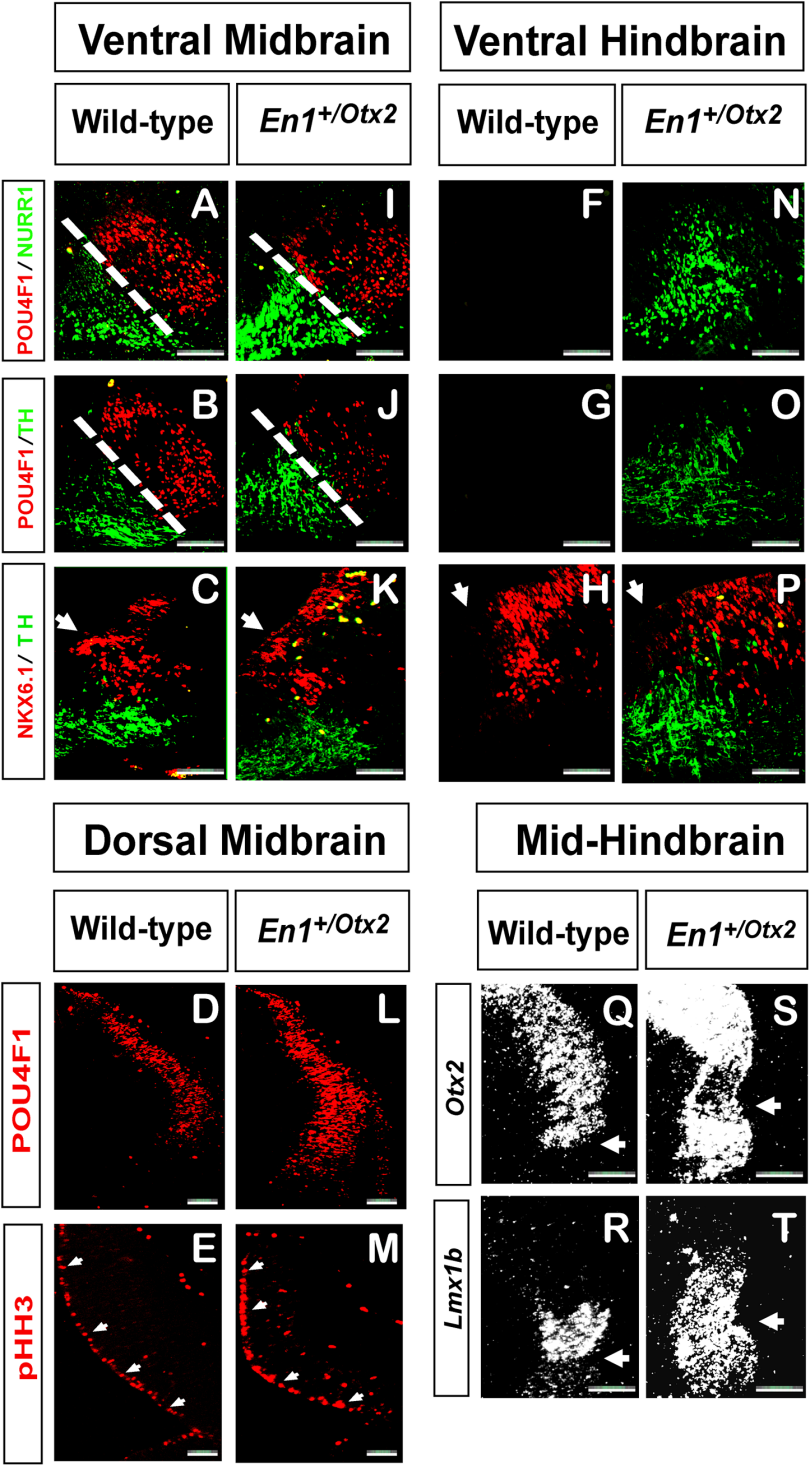
Overexpressing *Otx2* in the hindbrain leads ventrally to a specific expansion of the mdDA neuronal population. Representative coronal (A-P) and parasagittal (Q-T) sections of midbrain and hindbrain regions of WT (A-H, Q-R) and *En1* ^+/Otx2^ embryos (I-P, S-T). (A-C, I-K) In the ventral midbrain of WT and of *En1* ^+/Otx2^ mutants, NKX6.1 and the RN as visualized by POU4F1 are laterally adjacent to the mdDA neurons. (F-H) In the ventral hindbrain of WT mdDA markers are not observed and the NKX6.1 shows an expression pattern typical for the rostal hindbrain. (N-P) In the ventral hindbrain of *En1* ^+/Otx2^mutants the mdDA markers NURR1 and TH are present, but laterally not flanked by the RN or by the ventricular NKX6.1 expression domain (arrow). (D-E, L-M) In the dorsal midbrain of *En1* ^+/Otx2^embryos, POU4F1 and the proliferation marker PHH3 are increased. (Q, R) In WT, *Otx2* and *Lmx1b* expression extend until the isthmic constriction (indicated by arrow) marking the caudal border of the midbrain. In *En1* ^+/Otx2^ mutants the caudally extended *Otx2* expression domain leads to a concomitant caudal extension of the *Lmx1b* expression domain. (Scale bar, 100 μm).

Along the anterior-posterior axis of the midbrain in wild-type (WT) animals the RN, as visualized by POU4F1 expression, was located directly lateral to TH^+^ and NURR1^+^ mdDA neurons (Fig 1A and 1B). The RN and adjacent mdDA neurons were both posteriorly-extending up to the caudal border of the midbrain, but did not cross the boundary into the hindbrain (Fig 1F and 1G). In contrast, in *En1* ^+/Otx2^ mutants, the caudally-enlarged NURR1^+^ and TH^+^ cell population were not flanked by POU4F1^+^ cells laterally, indicating that the extended *Otx2* expression domain does not induce caudal extension of the RN (Fig 1N and 1O).

The phenotypic identity and location of the RN are controlled by the transcription factor NKX6.1[31]. In WTs directly adjacent to mdDA neurons, NKX6.1 was expressed in two domains, one in the ventricular zone above the mdDA neurons and one in the mantle zone (Fig 1C). In the hindbrain, the ventricular expression domain adjacent to the mantle zone expression area was missing (Fig 1H), in accordance with previously reported differences between the mid- and hindbrain NKX6.1 expression domain[32]. In *En1* ^*+/Otx2*^ mutants, there was no NKX6.1 expression in the ventricular zone above the caudally-extended mdDA neurons (Fig 1P). This further supports our contention that the posteriorly-extended mdDA neurons in *En1* ^+/Otx2^ mutants are actually located in the hindbrain.

To further characterize the differential effect of *Otx2* on the dorsal versus ventral midbrain formation, we visualized POU4F1 expression in the tectal midbrain of *En1* ^+/Otx2^ mutants. It was previously shown that *Otx2* specifies tectal fate independent of the MHO[33]. POU4F1 also plays a critical role in the formation of tectal neurons and their projections[34]. Nevertheless, the interaction of these two genes in tectal formation has been barely investigated. In contrast to the ventral midbrain, in which an increase in *Otx2* expression did not affect the number of POU4F1^+^ neurons, we found that POU4F1 was significantly increased in the dorsal midbrain of mutants (Fig 1D and 1L). In addition, mutants exhibited an upregulation of the mitosis marker, PHOSPHO-HISTONE H3 in this region (Fig 1E and 1M).

Taken together, we conclude that an early caudal expansion of *Otx2* under the endogenous *En1* promoter leads to a specific caudal extension of mdDA neuronal populations. These mutants, therefore, represent a useful tool to study the specific effects of *Otx2* on mdDA neuron development. In addition, our data suggest that by regulating the expression of *Pou4F1*, *Otx2* also affects tectal formation.

To further assess how *Otx2* and signals generated by the MHO interact in the formation of mdDA neurons, we investigated *Lmx1b* expression in *En1* ^+/Otx2^ mutants. We found that the expression domain of *Lmx1b* at E10.5 in *En1* ^+/Otx2^ mutants was caudally-extended to the same degree as the mdDA neuronal population, suggesting that *Otx2* might well control the formation of mdDA neurons via *Lmx1b* (Fig 1Q–1T).

### *Otx2* requires *Lmx1b* for the development of mdDA neurons

**Fig 2:**
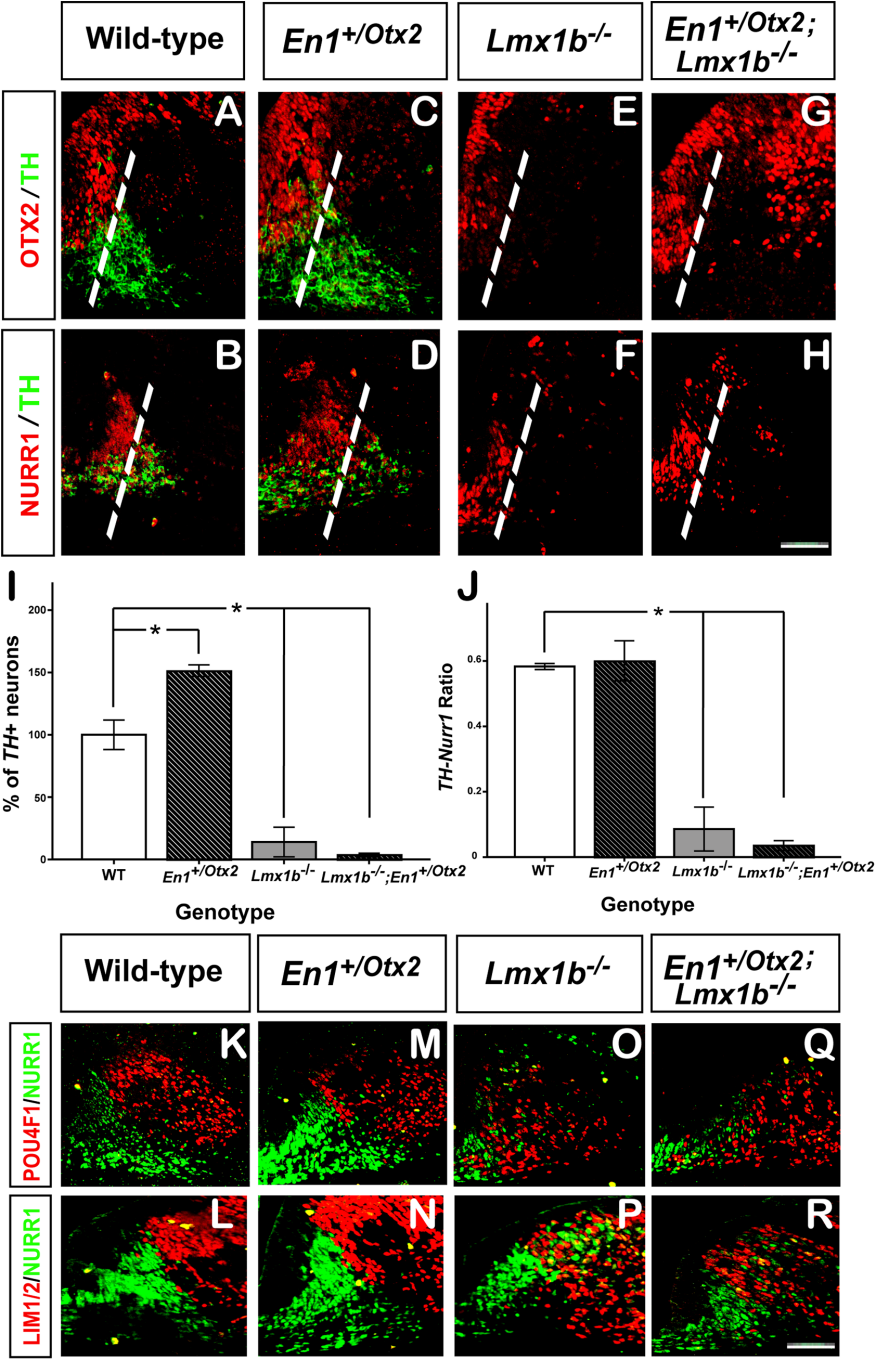
*Otx2* requires *Lmx1b* for the development of mdDA neurons. Representative coronal midbrain section of E12.5 WT (A-B, K-L), *En1* ^+/Otx2^(C-D, M-N), *Lmx1b* ^*-/-*^ (E-F, O-P) and *En1* ^+/Otx2^; *Lmx1b* ^*-/-*^ (G-H, Q-R) embryos. (I-J) Quantification of TH^+^ neurons and ratio of TH^+^-NURR1^+^neurons in different gentypes. (C-D) In *En1* ^+/Otx2^ embryos the number of TH^+^ and NURR1^+^ neurons is increased. (E, F) In *Lmx1b* ^*-/-*^ embryos, *Otx2* is downregulated and TH^+^ neurons are reduced in the medial region of the mdDA precursor domain and missing in the lateral domain (dotted line indicate border between medial and lateral region). (G, H) In *En1* ^+/Otx2^;*Lmx1b* ^*-/-*^ mutants *Otx2* is not sufficient to induce the terminal differentiation of NURR^+^TH^-^ cells to fully differentiated NURR^+^TH^+^ mdDA neurons and to rescue the lateral TH^+^ mdDA neurons. (I) Compared to the number of TH^+^ neurons in WT, the number of TH neurons in *En1* ^+/Otx2^ mutants are significantly increased and decreased in *Lmx1b* ^*-/-*^ and *En1* ^+/Otx2^; *Lmx1b* ^*-/-*^ embryos. (K, L) In WT there is a clear border between the mdDA neurons and the POU4F1 and LIM1/2 expression domain. (M, N) In *En1* ^+/Otx2^ mutants the border is maintained, but the mdDA neuronal population is expanded laterally, without affecting the size of the RN. (O, P) In *Lmx1b* ^*-/-*^, POU4F1^+^ and LIM1/2^+^ cells are mixed with NURR1^+^ neurons. (Q, R) In *En1* ^+/Otx2^;*Lmx1b* ^*-/-*^ mutants *Otx2* did not re-establish the lateral border of the mdDA precursor field. (Scale bar, 100 μm).

Interaction between *Otx2* and *Lmx1b* in the formation of mdDA neurons was assessed by following the expression of OTX2 in *Lmx1b* ^*-/-*^ mutants. At E12.5 a significant reduction of OTX2 was recorded in the ventral midbrain of *Lmx1b* ^*-/-*^ mutants (Fig 2A and 2E), suggesting that a downregulation of OTX2 could mediate the loss of mdDA neurons in *Lmx1b* ^*-/-*^.

At E12.5 a significant increase (51.3 ± 2.1%) in TH positive cells was noted in *En1* ^*+/Otx2*^ mutants compared to WTs (^*Otx2*^ *F*_1,8_ = 5.501, p = 0.047; Fisher’s LSD *post hoc* p = 0.003), consistent with previous reports[10] (Fig 2A, 2C and 2I). In *Lmx1b* ^*-/-*^ embryos the number of TH^+^ neurons were significantly decreased (82.1 ± 3.2%) compared to WTs (^*Lmx1b*^ *F*_1,8_ = 197.071, p<0.001; Fisher’s LSD *post hoc* p<0.001). To determine whether restoring OTX2 expression in *Lmx1b* ^*-/-*^ would reverse the various alterations observed in the mdDA neurons, we generated compound mutants in which *Lmx1b* was missing and *Otx2* was expressed under the control of the endogenous *En1* promoter (*En1* ^*+/Otx2*^ *;Lmx1b* ^-/-^). Despite the strong expression of OTX2 in compound mutants, there was no difference between the number of TH^+^ neurons between *En1* ^*+/Otx2*^ *;Lmx1b* ^-/-^ and *Lmx1b* ^*-/-*^ embryos (Fisher’s LSD *post hoc* p = 0.423) (Fig 1I). Moreover, the lateral population of mdDA neurons was missing in *En1* ^*+/Otx2*^ *;Lmx1b* ^-/-^, as in *Lmx1b* ^*-/-*^ (Fig 1E, 1F, 1G and 1H). The remaining medial mdDA neuronal populations exhibited alterations in their terminal differentiation as indicated by a significant reduction (84.0 ± 2.9%) in the number of NURR1^+^ cells expressing TH (^*Lmx1b*^ *F*_1,8_ = 131.122, p<0.001; Fisher’s LSD *post hoc* p<0.001) (Fig 2F, 2H and 2J). However, there was no significant difference in the number of NURR1^+^ cells expressing TH between *Lmx1b* ^*-/-*^ and the compound mutants (Fisher’s LSD *post hoc* p = 0.464). In WT and *En1* ^*+/Otx2*^ animals there was a sharp lateral border of the mdDA nuclei to the POU4F1 positive RN and LIM1/2 positive territory (K-N). In *Lmx1b* ^*-/-*^ the mdDA precursor domain intermingled with laterally-adjacent POU4F1 and LIM1/2 positive cells, which was not reversed by overexpression of *Otx2* in compound mutants (Fig 2O–2R). Taken together, we conclude that *Otx2* cannot compensate for the loss of *Lmx1b* in the development of mdDA neurons, suggesting that *Otx2* requires *Lmx1b* for the control of the formation of mdDA neurons.

### *Otx2* does not require *Lmx1b* for the induction of OMNs

To study whether expression of *Otx2* is sufficient to induce other ventral midbrain populations in the absence of *Lmx1b*, we studied the formation of OMNs as visualized by ISLET1 expression in *En1* ^*+/Otx2*^ *;Lmx1b* ^*-/-*^ mutants.

**Fig 3:**
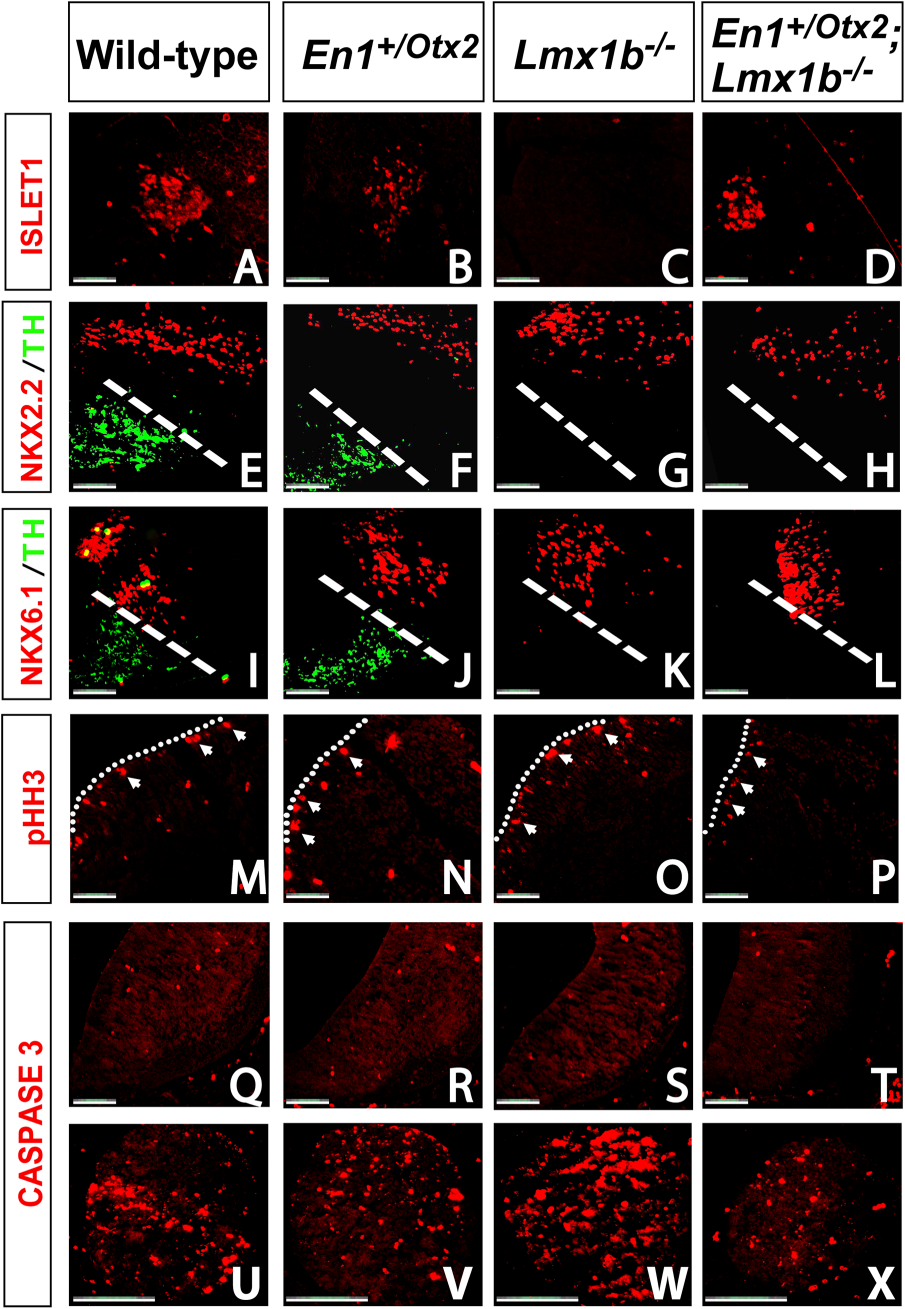
*Otx2* does not require *Lmx1b* for the induction of OMNs. Representative coronal midbrain section of E12.5 WT (A, E, I, M, Q, U), *En1* ^+/Otx2^ (B, F, J, N, R, V), *Lmx1b* ^*-/-*^ (C, G, K, O, S, W) and *En1* ^+/Otx2^;*Lmx1b* ^*-/-*^ (D, H, L, P, T, X) embryos. In WT (A) and *En1* ^+/Otx2^ (B) mutants OMNs are present as visualized by ISLET1. In *Lmx1b* ^*-/-*^ (C) ISLET1 expression is missing. In *En1* ^+/Otx2^;*Lmx1b* ^*-/-*^ (D) the restored *Otx2* expression leads to a significant rescue of OMNs, as indicated by ISLET1 expression. (E-H, I-L) In all genotypes the patterning of the ventral midbrain is unperturbed as indicated by normal expression of NKX2.2 and NKX6.1. (M-P) *En1* ^+/Otx2^ mutants show an increase in proliferation in the mdDA precursor domain as marked by an increase in PHH3. In contrast *Lmx1b* ^*-/-*^ and *En1* ^+/Otx2^;*Lmx1b* ^*-/-*^ mutants do not show any abnormalities in the expression of PHH3. (Q-T) Apoptosis as visualized by CASPASE 3 expression was normal in all genotypes. (U-X) Trigeminal ganglia showing strong CASPASE 3 expression were used a positive control. (Scale bar, 100 μm).

In *Lmx1b* ^*-/-*^ animals the number of ISLET1^+^ cells was reduced to 4.3 ± 1.2% of WTs (^*Lmx1b*^ *F*_1,21_ = 83.716, p<0.001; Fisher’s LSD *post hoc* p<0.001). In compound mutants *Otx2* lead to a significant increase of ISLET1^+^ neurons compared to *Lmx1b* ^*-/-*^ (^Otx2*Lmx1b^ *F*_1,21_ = 6.954, p<0.015; Fisher’s LSD *post hoc* p = 0.001) and to a recovery of 61.2 ± 2.7% of ISLET1^+^ neurons compared to WTs. The number of ISLET^+^ positive cells did not differ between *En1* ^*+/Otx2*^ mutants and WTs (Fisher’s LSD *post hoc* p = 0.691) (Fig 3A–3D).

Next we assessed the integrity of the ventral midbrain in mutants by visualizing the expression of patterning genes. We found that NKX2.2 (Fig 3E–3H) and NKX6.1 (Fig 3I–3L) were unaltered in all investigated genotypes. Moreover, we did not find any changes in mitosis as visualized by PHOSPHO-HISTONE H3 in the mdDA precursor domain (Fig 3M–3P) or apoptosis as visualized by activated CASPASE–3 (Fig 3Q–3T) in all genotypes at E12.5.

PHOSPHO-HISTONE H3^+^ cells in the mdDA progenitor domain were counted in WT, *En1* ^+/Otx2^, *Lmx1b* ^*-/-*^ and *En1* ^*+/Otx2*^ *;Lmx1b* ^*-/-*^ embryos. A two-way ANOVA of the ratio between the PHOSPHO-HISTONE H3^+^ cells revealed no significant difference between any of the groups, with a trend increase in *En1* ^*+/Otx2*^ mutants (^*Lmx1b*^ *F*_1,21_ = 0.514, p = 0.497; ^*Otx2*^ *F*_1,21_ = 5.206, p = 0.056). Since virtually no CASPASE–3 staining was observed in any of the genotypes a quantification was not performed. The trigeminal ganglion showing strong CASPASE–3 staining was used as a positive control (Fig U-X) [35].

In order to assess whether changes in mitosis or apoptosis are present at earlier developmental time points we also studied PHOSPHO-HISTONE H3 and CASPASE–3 at E11.5. There was a significant increase in the number of PHOSPHO-HISTONE H3 positive cells in *En1* ^*+/Otx2*^ (31.0 ±1.8%) compared to WT animals (^*Otx2*^ *F*_1,16_ = 11.281, p = 0.004; Fisher’s LSD *post hoc* p = 0.004). *Lmx1b* ^*-/-*^ embryos and compound mutants did not show significant differences compared to WTs in the number of PHOSPHO-HISTONE H3 positive cells (^*Lmx1b*^ *F*_1,16_ = 1.439, p = 0.248; Fisher’s LSD *post hoc* p = 0.867 and p = 0.146). As for E12.5, the very few CASPASE–3 cells did not allow quantification. We conclude that in contrast to mdDA neurons, *Otx2* does not require *Lmx1b* for the induction of OMNs.

### In *Lmx1b* ^*-/-*^ embryos NURR1^+^ neurons express LMX1A and FOXA2 but not EN1

**Fig 4:**
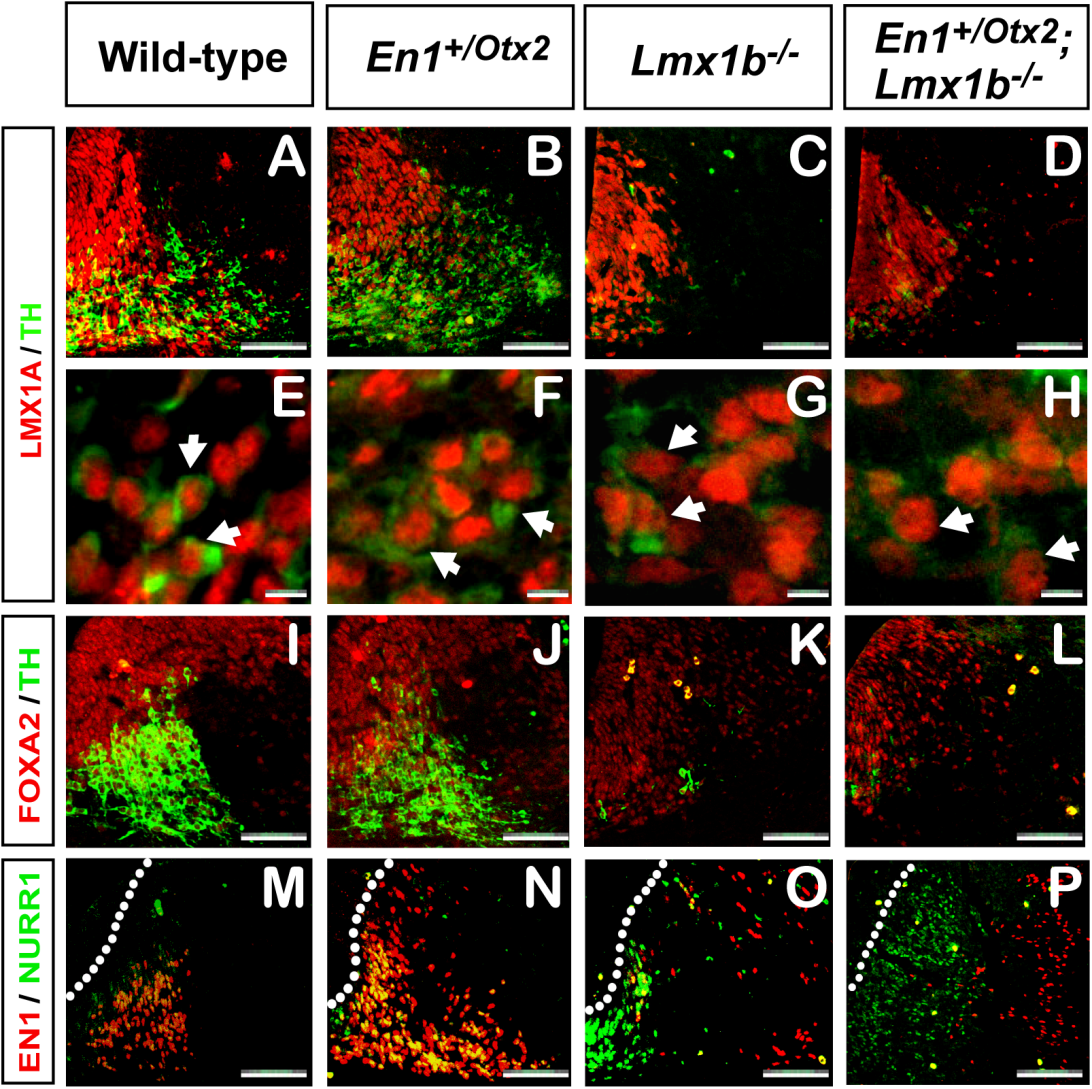
In *Lmx1b* ^*-/-*^ embryos NURR1^+^ neurons express LMX1A and FOXA2 but not EN1. Representative coronal midbrain section of E12.5 WT (A, E, I, M), *En1* ^+/Otx2^(B, F, J, N), *Lmx1b* ^*-/-*^ (C, G, K, O) and *En1* ^+/Otx2^; *Lmx1b* ^*-/-*^(D, H, L, P) embryos. LMX1A (A-H) and FOXA2 (I-L), which are important for the formation of the mdDA precursor domain do not show any changes in expression in *Lmx1b* ^*-/-*^ or in *En1* ^+/Otx2^;*Lmx1b* ^*-/-*^ embryos. In contrast EN1 (O) is lost in NURR1^+^ neurons in *Lmx1b* ^*-/-*^ mutants. In *En1* ^+/Otx2^;*Lmx1b* ^*-/-*^ embryos (P), *Otx2* is not sufficient to rescue the EN1 expression lost in *Lmx1b* ^*-/-*^. (Scale bar, A-D, I-P, 100 μm, E-H 6 μm).

We next studied in each of the different genotypes, the expression of the transcription factors *Lmx1a*, *Foxa2* and *En1*, which all play central roles in the development of mdDA neurons[4][14][15][36]. We observed unaltered expression of LMX1A (Fig 4A–4D) and FOXA2 (Fig 4I–4L) in the ventral midline of all mutants. Double immune labeling with LMX1A and TH antibodies indicated that the few remaining TH^+^ cells in *Lmx1b* ^*-/-*^ and compound mutants were all LMX1A^+^ (Fig 4C, 4D, 4G and 4H).

*En1* is expressed during embryogenesis at the MHO and in developing mdDA neurons. Together with *En2*, it is essential for the formation of the mature mdDA phenotype and for the survival of these neurons[14][37][38][39]. Interestingly, *En1* ^*-/-*^ phenocopies important aspects of the mdDA phenotype of *Lmx1b* ^*-/-*^, suggesting that *Lmx1b* and *En1* are active in the same pathway directing the development of mdDA neurons[38]. However, the signals inducing and maintaining *En1* expression in the mdDA precursors are largely unknown. To address this issue, we followed EN1 expression in the various genotypes. In WTs and in *En1* ^*+/Otx2*^ embryos, all NURR1 expressing cells co-expressed EN1 (Fig 4M and 4N). In contrast, in *Lmx1b* ^-/-^ and *En1* ^+/Otx2^;*Lmx1b* ^*-/-*^ mutants EN1 expression was lost in NURR1 expressing cells, while EN1 expression was maintained and possibly even augmented in the region adjacent to the NURR1^+^ mdDA neurons (Fig 4O and 4P). We conclude that in contrast to LMX1A and FOXA2, EN1 expression is dependent on *Lmx1b* in NURR1^+^ neurons and cannot be substituted for by *Otx2*.

### *Wnt1* and *Fgf8* expression is induced but not maintained in *Lmx1b* ^-/-^ embryos

In order to better understand how *Lmx1b* directs the formation of mdDA neurons via the MHO, we employed *Lmx1b* ^-/-^ embryos to study *Fgf8*, *Wnt1* and *Otx2* expression, each of which is critical for MHO positioning, maintenance and activity. Highly sensitive radioactive mRNA *in situ* hybridization was performed on sagittal paraffin-embedded tissue sections taken from the mesencephalic flexure, parasagittal region of the MHO and from the dorsal (tectal) region of the MHO.

**Fig 5:**
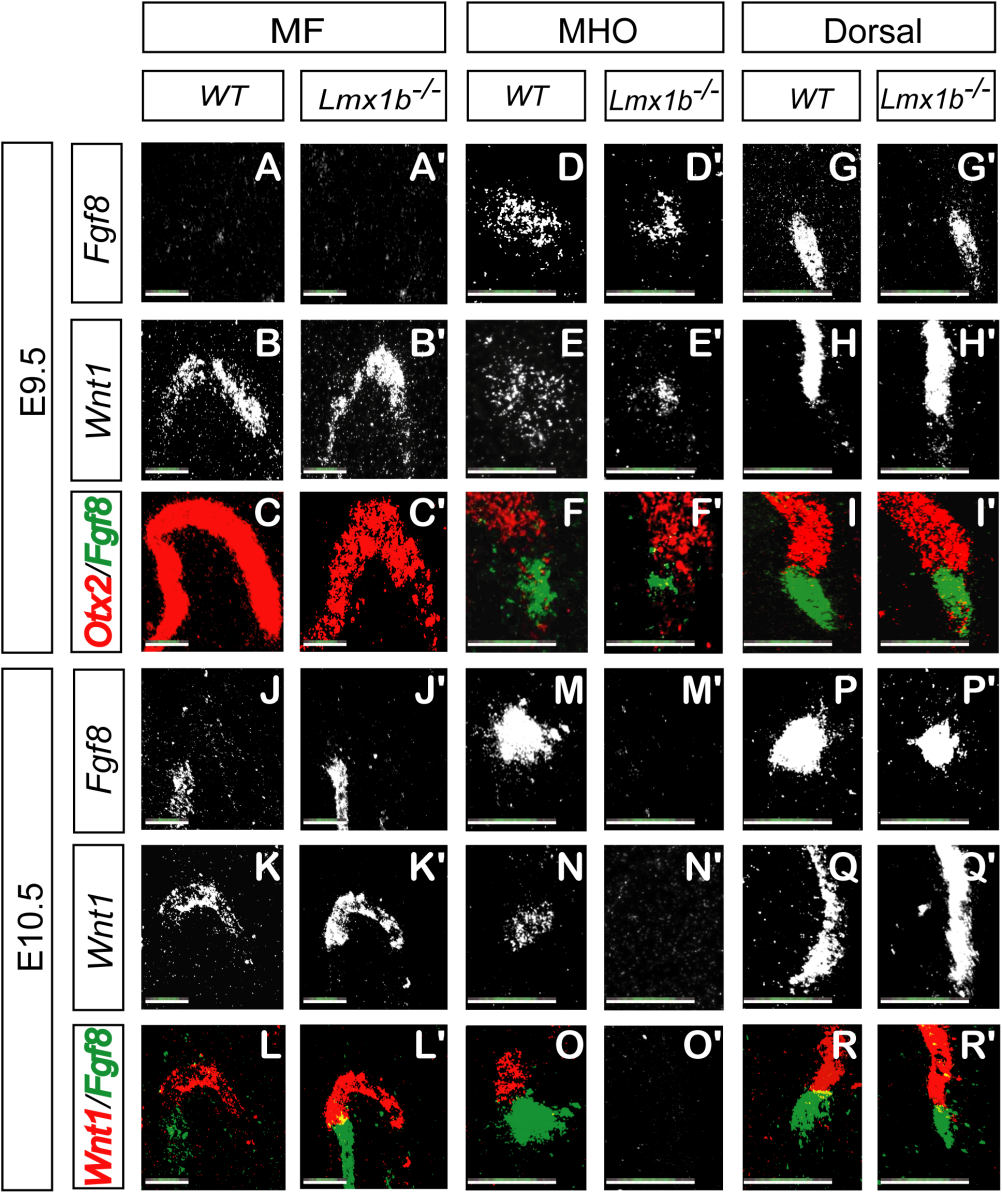
*Wnt1* and *Fgf8* expression is induced but not maintained in *Lmx1b* ^*-/-*^ embryos. Representative sagittal sections of the mesencephalic flexure (A-C, A'-C', J-L, J'-L') and the lateral (D-F, D'-F', M-O, M'-O') and dorsal (G-I, G'-I', P-R, P'-R') aspect of the MHO of E9.5 (A-I, A'-I') and E10.5 (J-R, J'-R') WT (A-R) and *Lmx1b* ^*-/-*^ (A'-R') embryos. Genes were visualized by radioactive mRNA *in situ* hybridization. (B, B') At E9.5 *Wnt1* expression is normally induced in the MF of *Lmx1b* ^*-/-*^. (D'-E', G'-H') At E9.5, *Fgf8* and *Wnt1* expression are reduced but present in *Lmx1b* ^*-/-*^ embryos at the lateral and dorsal aspect of the MHO. (C, C', F, F', I, I') Overlay of adjacent sections indicate that *Fgf8* is expressed in *Lmx1b* ^*-/-*^ as in WT directly posterior to the *Otx2* expression domain. (K, K') At E10.5 *Wnt1* expression is maintained at the MF of *Lmx1b* ^*-/-*^. (M'-N', P'-Q') At E10.5, expression of Fgf8 and Wnt1 are lost in the lateral MHO domain, but still present in the dorsal region of the MHO. (L-R, L'-R') Overlay of adjacent sections indicate that *Fgf8* is expressed in *Lmx1b* ^*-/-*^ as in WT directly posterior to the *Wnt1* expression domain. (Scale bar, 250 μm).

At E9.5, in the mesencephalic flexure of WTs and *Lmx1b* ^-/-^ mutants, *Fgf8* was not expressed (Fig 5A and 5A'). *Wnt1* expression did not differ between mutants and WTs (Fig 5B and 5B'). As with immunohistochemistry (Fig 2A and 2E), in the ventral midbrain of *Lmx1b* ^-/-^embryos, *Otx2* transcription was reduced (Fig 5C and 5C'). In parasagittal and dorsal sections of the MHO, *Fgf8* as well as *Wnt1* expression was detected in *Lmx1b* ^-/-^embryos (Fig 5D, 5D', 5E, 5E', 5G, 5G', 5H and 5H'). While parasagittal expression levels were reduced, dorsal expression in mutants was similar to WTs. In order to determine if the lack of *Lmx1b* affects the normal positioning of *Fgf8* expression at the MHO, we compared the expression domain of this growth factor to the *Otx2* expression area on consecutive sections (Fig 5F, 5F', 5I and 5I'). As seen in WT and also in sections from *Lmx1b* ^-/-^ animals, the *Fgf8* expression domain was directly caudal to the *Otx2* expression domain, suggesting that the decreased *Fgf8* signal is properly positioned along the anterior-posterior axis.

At E10.5, sections from *Lmx1b* ^-/-^ embryos exhibited a normal *Wnt1* expression at the mesencephalic flexure (Fig 5K and 5K'). A short exposure of the slides to autoradiography films, used for the quantification of in situ hybridization experiments, did not show any differences in signal intensity between WTs and mutants. This suggests that the lack of apparent difference between signal strength in WTs and mutants seen on the slides is unlikely caused by an overexposure of the slides to the dipping solution which would prevent the reaction to be in the linear range. *Fgf8* and *Wnt1* expression was not detected at the parasagittal region of the MHO (Fig 5M' and 5N'). In contrast, both *Fgf8* and *Wnt1* dorsal expression domains were present at this embryonic stage (Fig 5P' and 5Q').

In WTs, *Wnt1* and *Fgf8* expression at the MHO are reciprocally maintained[40]. *Wnt1* is expressed in the caudal midbrain, while *Fgf8* expression is found directly adjacent, in the rostral hindbrain[29]. In order to test whether the dependency of these two genes are maintained in *Lmx1b* ^-/-^ mutants, we compared their area of expression. Consecutive sections, hybridized with *Wnt1* and with *Fgf8* probes, indicate that *Fgf8* is expressed in WT as well as in mutants directly caudal to the *Wnt1* domain (Fig 5L, 5L', 5O, 5O', 5R and 5R'). While the dorsal *Fgf8* domain was no longer detected by E11.5 the dorsal *Wnt1* domain was lost by E12.5 (data not shown). Taken together, *Lmx1b* is required for the maintenance of *Fgf8* and *Wnt1* in a ventro-dorsal gradient, but not for the induction of these two morphogens.

### *Lmx1b* ^*-/-*^ embryos show a specific spatio-temporal loss of *En1* and *Wnt1* expression

**Fig 6:**
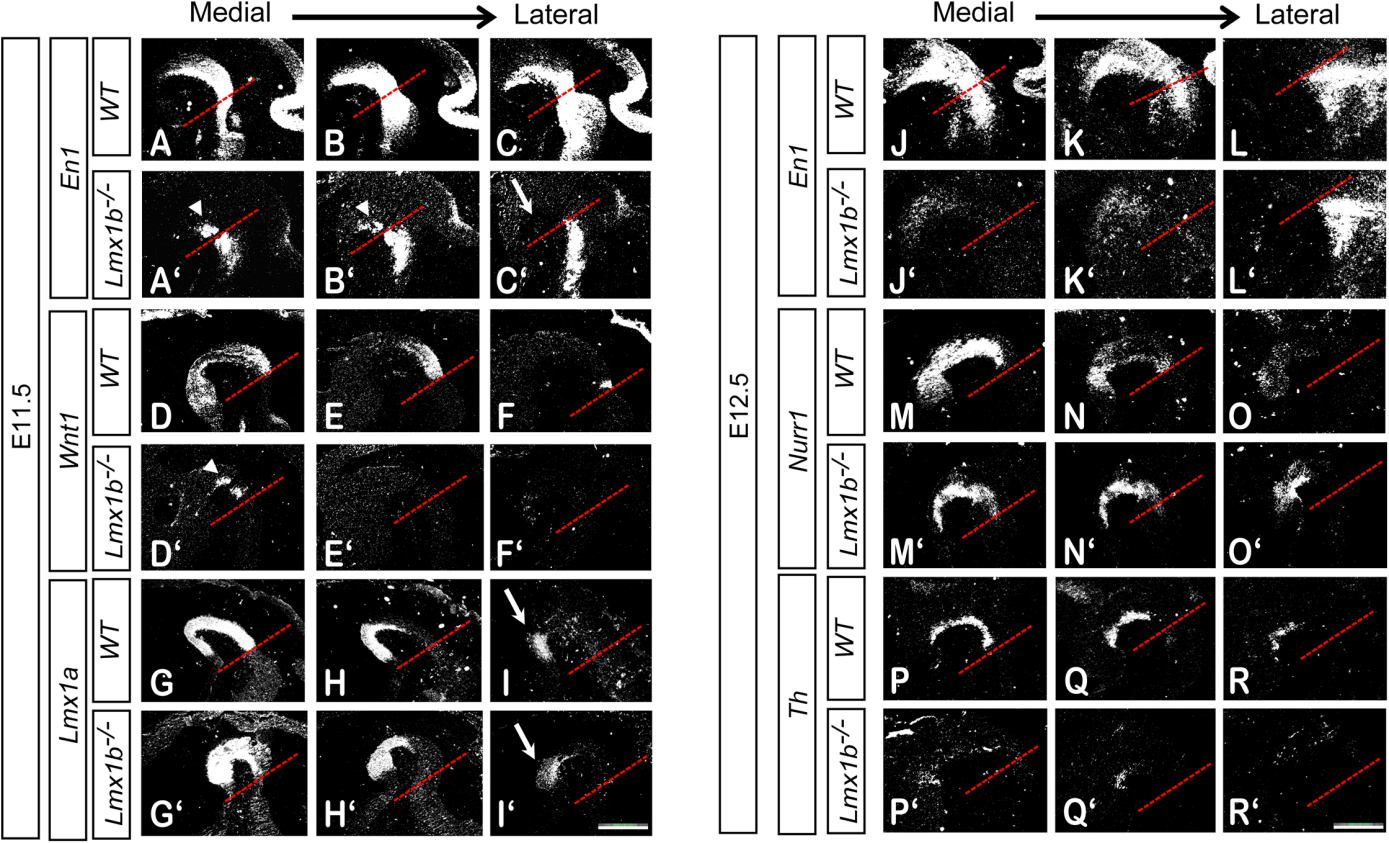
*Lmx1b* ^*-/-*^ embryos show a specific spatio-temporal loss of *En1* and *Wnt1* expression. Representative sagittal sections of the mesencephalic flexure of WT (A-R) and *Lmx1b* ^*-/-*^ (A'-R') embryos at E11.5 (A-I, A'-I') and E12.5 (J-R, J'-R'). Genes were visualized by radioactive mRNA *in situ* hybridization. (A-F, A'-F') At E11.5, *En1* and *Wnt1* expressions are lost in *Lmx1b* ^*-/-*^ embryos at the rostrolateral aspect of the mesencephalic flexure (arrow) and only the caudomedial expression domain is detectable (arrow head). (G-I, G'-I') The expression of *Lmx1a* is similar between the WT and the *Lmx1b* ^*-/-*^, including the rostrolateral domain (arrow). (J-L, J'-L') At E12.5, the caudomedial *En1* expression domain is lost in *Lmx1b* ^*-/-*^, while the hindbrain *En1* expression is still present. (P-R, P'-R') *Th* expression is significantly reduced. (M-O, M'-O'). No significant difference was found in the expression of *Nurr1*. Red dotted line represent boundary between midbrain and hindbrain. (Scale bar, 250 μm).

We next followed the expression pattern of genes associated with the formation of mdDA neurons in *Lmx1b* ^-/-^ embryos at E11.5 and E12.5. We employed radioactive *in situ* hybridization to visualize gene transcripts on series representing the mesencephalic flexure, along the medial to lateral extension. In *Lmx1b* ^-/-^ at E11.5, *En1* expression was reduced in the caudomedial domain and absent in the rostrolateral domain (Fig 6A'–6C'). At E12.5, *En1* was absent from the entire mesencephalic flexure (Fig 6J'–6L'). In *Lmx1b* ^-/-^ at E11.5, *Wnt1* expression was significantly reduced, with only a small region of expression visible in the caudomedial region, and virtually absent at E12.5 (Fig 6D'–6F', data not shown). *Th* expression was reduced in *Lmx1b* ^-/-^ mutants at E12.5 (Fig 6P'–6R'), and not detected at around E17.5, as previously reported by Smidt et al. (data not shown). *Lmx1b* ^-/-^ embryos did not exhibit any apparent changes in expression of *Lmx1a* (Fig 6G'–6I') or of *Nurr1* (Fig 6M'–6O') at either age. Taken together, the inactivation of *Lmx1b* leads to a specific spatio-temporal loss of *En1* and *Wnt1* expression.

## Discussion

### Overexpressing *Otx2* in the hindbrain leads ventrally to a specific expansion of the mdDA neuronal population

In dorsal brain stem of *En1* ^+/Otx2^ mutants ectopic expression of *Otx2*, induces a general expansion of all midbrain structures at the expense of the rostral hindbrain[29]. In contrast, in the ventral brain stem of *En1* ^+/Otx2^ mutants, *Otx2* expression results in a specific expansion of the mdDA neuronal population. The molecular mechanisms mediating these differential effects of *Otx2* on the development in dorsal and ventral parts of the brain stem are unknown. The mutual repression of midbrain *Otx2* and hindbrain *Gbx2* expression is instrumental in defining midbrain versus hindbrain fate[29][41]. Loss of function experiments for *Otx2* as well as *Gbx2* indicate however that this antagonizing interaction is less apparent in the ventral brain stem[32][42]. As a consequence, overexpression of *Otx2* in the dorsal brainstem could lead to a general expansion of all dorsal midbrain structures at the expense of the rostral dorsal hindbrain. In the ventral brain stem, *Otx2* was previously shown to antagonize *Nkx2*.*2*, critically defining the border between the mdDA precursor domain and the adjacent serotonergic precursor domain[6]. Together, these data suggest that the general changes described in the present study for the dorsal hindbrain reflect a mutual repression of *Otx2* and *Gbx2*, whereas the specific changes in the ventral hindbrain seem to be based on the antagonism of *Otx2* and *Nkx2*.2.

### *Otx2* requires *Lmx1b* for the development of mdDA neurons

*Otx2* has been shown to play a critical role at different stages of mdDA neuron development[11][21][22][23][32]. The role of *Otx2* in the terminal differentiation of mdDA neurons, however, remains poorly understood. Our data provide evidence that *Otx2* requires *Lmx1b* activity to induce NURR1^+^TH^+^ neurons from immature NURR1^+^TH^-^ precursors. The molecular pathway by which *Lmx1b* regulates the final step is as yet unknown. Similar to *Lmx1b* ^*-/-*^ mutants, NURR1^+^TH^-^ neurons fail to fully differentiate to NURR1^+^TH^+^ mdDA neurons in *Foxa1*/2 mutants[15], suggesting that *Lmx1b* could induce the terminal differentiation of mdDA neurons by regulating *Foxa2* expression. However, this hypothesis is not supported by our findings since *Foxa2* expression is not altered in *Lmx1b* ^*-/-*^ mutants. Since *Lmx1b* is also normally expressed in *Foxa2* mutants, it is clear that *Lmx1b* is also not downstream of *Fox2a* in this cascade.

*Otx2* and *Lmx1b* have both been implicated in defining the lateral border of the mdDA precursor field. Thus, overexpressing *Otx2* in the *En1* expression domain leads to a lateral expansion of the *Lmx1b* expression area and concomitant mdDA precursor field[21]. In contrast, *Lmx1b* ^-/-^ embryos are characterized by an invasion of POU4F1 positive RN cells into the mdDA precursor domain[24]. Analysis of *Lmx1b* ^-/-^ mutants and embryos ectopically expressing *Sim1* in the mdDA precursor domain indicates that an antagonism between *Lmx1b* and *Sim1* is important for defining the lateral border of the mdDA neuronal population to that of the RN[24][43]. An antagonism between *Otx2* and *Sim1* has not been established in this context. Based on the results of our compound mutants, it appears likely that *Otx2* modulates the medio-lateral boundary of mdDA nuclei by regulating *Lmx1b*, which in turn represses *Sim1* and the lateral red nucleus identity.

### The role of *Otx2* and *Lmx1b* in OMNs formation

In contrast to its effect on mdDA neurons, *Otx2* can induce the formation of OMNs independent of *Lmx1b*. *Lmx1b* affects the development of OMNs by inducing *Phox2a* expression and repressing *Sim1* transcripts required for formation of the adjacent RN[24]. The conditional inactivation of *Otx2* in the *En1* domain leads to a hypoplasia of OMNs, indicating that these neurons are dependent upon *Otx2*[32]. The down regulation of *Otx2* in *Lmx1b* ^-/-^ embryos and the rescue of OMNs in compound mutants suggest that *Lmx1b* operates upstream to or independent of *Otx2* in the specification of OMNs.

### The role of *Lmx1b* in controlling *Fgf8*, *Wnt1 and En1* expression at the MHO and mdDA progenitor domain

The conditional inactivation of *Lmx1b* using a *Shh*-Cre driver results in normal development of mdDA neurons. This indicates that *Lmx1b* regulates mdDA development not cell autonomously, but via the MHO[25]. *Fgf8*, *Wnt1* and *En1* are key elements of the MHO. Moreover, there is a dynamic spatio-temporal requirement of these genes in the development of mdDA neurons. Therefore, it is essential to study their expression in *Lmx1b* ^*-/-*^ embryos in order to understand how *Lmx1b* directs the formation of mdDA neurons.

Using whole-mount *in situ* hybridization, it was reported that *Fgf8* is not expressed in *Lmx1b* ^-/-^ embryos, indicating that *Lmx1b* is absolutely required for the induction of *Fgf8* expression and MHO activity[28]. However, since mdDA neurons are formed in *Lmx1b* ^-/-^mice, their lack of *Fgf8* would contradict previous findings that *Fgf8* is required for mdDA neuron induction[5]. Our finding that *Fgf8* is induced in *Lmx1b* ^-/-^mutants does not support the hypothesis that mdDA neurons can be induced in the absence of *Fgf8*. It seems possible to us that the discrepancy between our results and previous analysis of *Lmx1b* ^*-/-*^ embryos can be related to the higher sensitivity of radioactive *in situ* hybridization used in our study.

In *Lmx1b* ^-/-^ embryos and in conditional mutants using an *En1*-Cre driver to inactivate *Lmx1b*, *Wnt1* expression is dramatically reduced at the MHO during early somitogenesis, as well as in the ventral midbrain at E11.5 [24][27][28]. However, it is not known if *Lmx1b* is required for the induction of *Wnt1* at the mdDA precursor domain. Our finding that *Wnt1* is expressed normally in the ventral midbrain in *Lmx1b* ^-/-^, indicates that *Lmx1b* is not necessary for the induction of *Wnt1* expression, but rather for its maintenance. *Wnt1* has been shown to regulate the expression of *Otx2* [6][9]. It is therefore, conceivable that the reduction of OTX2 that we observed at E12.5 in *Lmx1b* ^*-/-*^ is mediated by the loss of *Wnt1*.

At the MHO of *Lmx1b* ^-/-^ mutants, expression of *En1* is initially induced but then lost at E9.5[28]. However, expression of *En1* in the mdDA precursor domain in these mutants has not been assessed. Based on the loss of *Pitx3* in *Lmx1b* ^-/-^[12] and the fact that *Pitx3* represses *En1* expression in the rostrolateral domain[44], upregulation of *En1* was anticipated in this area in *Lmx1b* ^-/-^. Unexpectedly, we observed downregulation of *En1* in the rostrolateral domain of these mutants, suggesting that *Lmx1b* regulates expression of *En1* in a *Pitx3* independent manner. The fact that in *Lmx1b* ^-/-^ mutants NURR1^+^ neurons, lack *En1*, in contrast to other genes important for formation of mdDA neurons, including *Foxa2* and *Lmx1a*, suggests that *Lmx1b* has differential effects on the gene regulator pathways in dopaminergic precursors.

In summary, our study demonstrates that *Otx2* is critically dependent on intact *Lmx1b* gene activity to direct the formation of mdDA neurons, but not for the generation of OMNs. In addition, *Lmx1b* regulates the spatio-temporal expression of *Fgf8*, *Wnt1* and *En1*, which are critical for the MHO function and mdDA development.
